# Exploring Hygienic Milk and Meat Handling Practices and Their Associated Risk Factors in and Around Ambo and Guder Towns, Ethiopia

**DOI:** 10.1155/ijfo/3064650

**Published:** 2025-09-25

**Authors:** Beka Ararsa Desisa, Birhan Agmas Mitiku

**Affiliations:** ^1^ School of Veterinary Medicine, Bahir Dar University, Bahir Dar, Ethiopia, bdu.edu.et; ^2^ Faculty of Veterinary Medicine and Animal Science, Federal University of Mato Grosso do Sul, Campo Grande, Brazil, ufms.br

**Keywords:** Ambo town, Guder town, handling practice, meat, milk

## Abstract

Food safety is crucial for life and health, as unsafe food causes a range of diseases from diarrhea to cancer. Poor food handling can lead to disease and malnutrition, affecting infants, young children, the elderly, and the sick. Meat and milk products are particularly susceptible, and poor hygiene conditions and lack of personal hygiene contribute to contamination. Thus, the objective of this study was to assess hygienic milk and meat handling practices and their associated risk factors along the value chain handlers in the study area. A cross‐sectional study survey was taking place from January to August 2024. A structured pretested questionnaire data collection tool was used to collect the data. A total of 186 respondents, found in the Ambo and Guder towns along the milk and meat value chain, were involved in the study. The data was analyzed using SPSS software Version 27. The study revealed that the hygienic milk and meat handling practices level of milkers, milk sellers, butchers, and meat sellers were 52.8%, 43.3%, 50%, and 54.3%, respectively. Respondent’s age and experience year were found to be statistically significant (*p* < 0.05) and showed good milk and meat handling practices. In conclusion, this study indicated that nearly half of the respondents had poor practices of milk and meat handling. This implies that the meat and milk products in the area may have public health risks. Therefore, creating and raising awareness about hygienic milk and meat handling practices in the study area is needed.

## 1. Introduction

Strong human–animal–environment relationships in developing countries trigger attention for food safety and quality. Increased human demand for animal products necessitates safe and sustainable practices, which leads to disease avoidance and quality maintenance. According to FAO, a One Health approach is crucial to addressing food safety concerns. Commercial considerations include safeguarding consumer trust in safe and high‐quality animal products, with long‐term food safety and environmental health relying on sustainable livestock production [1–3].

Food contamination may occur at any point during the supply chain, impacting food safety. Every food producer, shipper, processor, distributor, and food handler plays a part in maintaining food safety. Preslaughter or preprocessing risks can be caused by contaminated water, unsanitary animal handling, unclean facilities, and cross‐contamination [4–6]. Inappropriate handling of food in the home environment can occur during the pre‐preparation, preparation, and storage of food products [7, 8]. Some factors relate to the handler, such as age, education level, type of employment, and lack of prior knowledge, which directly affect hygienic–sanitary conditions during food preparation [9–11].

The food handler’s health status and hygiene practice are the foremost determinants of food contamination. This is a fact: food handler’s hygiene practices and health status are significant variables in determining the risk of food contamination because food handling by them can introduce pathogenic microorganisms into food, leading to food poisoning through absence of protective controls, unhygienic handling, cross‐contamination, or carriage of their own microorganisms. This can result in quality deterioration, economic losses, and public health concerns [12–15].

An estimated 600 million people worldwide become victims of foodborne illness every year, resulting in 420,000 deaths. Children under the age of 5 years are at greatest risk, and 125,000 deaths occur every year. The WHO estimates that low‐ and middle‐income countries incur a staggering economic burden, losing $110 billion annually in productivity and healthcare expenditures due to foodborne infections [16]. This happens due to the absence of proper care during production and processing, which leads to a serious public health risk, likely exposure to zoonotic pathogens that are transmitted between animals and humans [17, 18].

Of a range of disease problems, foodborne diseases are recognized to regularly occur and are related to low‐income countries, probably due to improper food handling, hygiene, lack of food safety laws and weak implementation systems, lack of economic assets to procure safety tools, and lack of education and/or training for different food handlers [19–22]. In Ethiopia, animal‐based meals like meat and milk are primarily responsible for causing food‐borne illness due to inappropriate handling, inadequate food safety policy, inadequate regulatory systems, and inadequate training of food handlers [6, 23, 24].

To our knowledge, the number of studies on hygienic milk and meat handling practices along the value chain in the study area is scanty. Both acquisition of food handling knowledge and hygienic food handling practice are critical; however, observing food handling practices is more crucial than knowledge for assessing food safety risks because individuals may have good knowledge but still engage in unsafe practices. Poor hygiene practices are largely responsible for foodborne outbreaks, and enhancing food handling practices by addressing the main factors can be more effective than merely enhancing knowledge alone. Several studies show that food handlers usually know the basics of food safety, but due to different factors, do not always put that knowledge into practice whenever they work [25–29]. Hence, assessing hygienic milk and meat handling practice level and its associated risk factors along the value chain of handlers in the study area was of supreme importance in order to provide useful data for future tailor‐made intervention. This work result will aid in the development of appropriate prevention strategies for milk and meat product foodborne diseases.

## 2. Methods and Materials

### 2.1. Study Area

**Figure 1:**
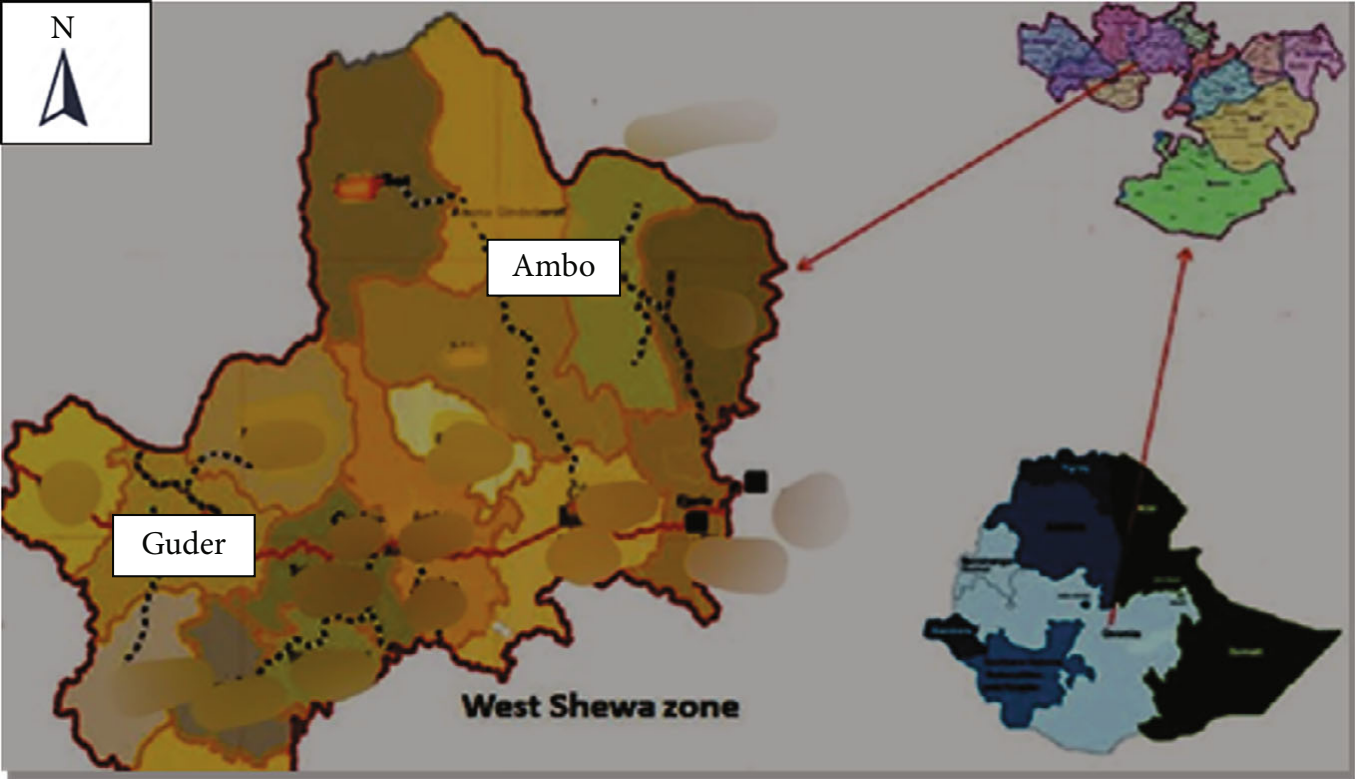
Geographic location of the study area (*source:* Modified map from spatial data in DIVA‐GIS 2020).

This study was taking place in Ambo and Guder towns of the West Shewa Zone, Oromia, Ethiopia (Figure 1). The towns were located 126 and 114 km to the west of Addis Ababa, respectively. The latitude and longitude of the towns were 8°59 ^′^N 37°51 ^′^E and 8°58 ^′^N 37°46 ^′^E with elevations of 2101‐m above sea level and temperatures ranging from 10°C to 29°C and 15°C to 29°C, respectively. The mean annual temperature of the towns over 30 years is about 18.64°C. The mean annual rainfall of the Ambo and Guder towns over 30 years is about 968.7 mm. The total number of cattle in and around the towns of Ambo and Guder is 144,243 and 174,799, respectively, and the present total populations of the towns are 70,900 and 21,700, respectively [30, 31].

### 2.2. Study Design

A community‐based cross‐sectional study design among milk and meat handlers and sellers was used for this study. It was conducted from January to August 2024.

### 2.3. Study Population

All smallholders and livestock farms, milk and meat shops, and municipal abattoirs found in Ambo and Guder towns were considered as the study population.

### 2.4. Inclusion and Exclusion Criteria

Inclusion criteria: A person (> 18 years of age) who worked as small holder’s farms, in milk shops, or in two municipal abattoirs was included in the study.

Exclusion criteria: A person who resided on smallholder farms, worked in milk shops, or worked in municipal abattoirs but was highly ill and a person unable to give the necessary information at the time of data collection was excluded from the study.

### 2.5. Sample Size Determination and Sampling Method

#### 2.5.1. Sample Size Determination

Sample size was determined using the formula by Yamane [32] that is used for known population. Our preassessment included nearly 348 permanently employed workers (milkers, milk sellers, butchers, and meat salers) in two study towns. Accordingly,

n=N/1+Ne2,

where *n* is the required sample size, *N* is the study population, and *e* is the error of margin; for the study whose confidence level is 95%, *e* = 5*%*. Thus, the overall sample size was 186 participants.

#### 2.5.2. Sampling Method

**Figure 2:**
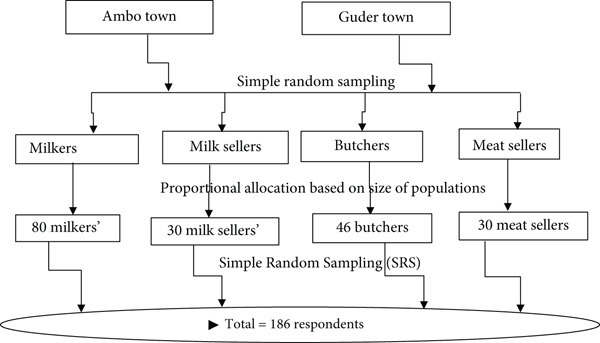
Diagrammatic presentation of study participant selection.

The total number of animals’ data in the study area from smallholder farmers, farms, milk shops, municipal abattoirs, and butcher houses from both towns was collected from the office of administration of Ambo and Guder towns. The simple random sampling method was used to choose respondents. In Ambo and Guder towns and their surrounding kebeles, participants were selected randomly. Of each study site, smallholders and farms (61 and 19), milk shops [30], two municipal abattoirs (Ambo 24 and Guder 22), and meat shops [30] from both towns, for a total of 186, were selected based on the inclusion and exclusion criteria stated above. The number was allocated based on proportionality to the size of the population (Figure 2).

### 2.6. Variables of the Study

#### 2.6.1. Dependent Variables

The dependent variables include hygienic meat and milk handling practice (good vs. poor practice).

#### 2.6.2. Independent Variables

Sociodemographic variables: Respondents’ age, sex, residence, and educational status were the variables considered as sociodemographic variables by this study.

Behavioral variables include the role/the value chain they work in, years of experience, and food handling training of participants.

Environmental variables include the respondent’s environmental hygiene and housing system (source of water and separated milking room).

### 2.7. Operational Definitions

Hygienic meat and milk handling practice level: which resulted in a response of either, choose the correct answer or wrong answer for each question. The number of questions for which the respondent gives correct responses was counted and scored. The score was then pulled together, and the mean score was computed to determine the overall practice of respondents. The study participants who correctly responded to greater than or equal to the mean score were said to have a good practice from those greater than or equal to the 12 practice questions provided.

The scoring of practice items was conducted in a straightforward manner, where a score of 1 was assigned for a response that demonstrated positive practice or a correct answer, while a score of 0 was given for any other response, including each instance of “*Do not know*” or an incorrect answer. The total score would indicate a positive practice scale if it exceeded 50%, or a negative practice score if it fell below 50%. This implies that good practice is defined as follows: for milkers, answering more than 10 out of 19 questions correctly; for milk sellers, answering more than six out of 11 questions correctly; for abattoir workers, answering more than six out of 12 questions correctly; and for meat sellers, answering more than six out of 12 questions correctly. The overall assessment of hygienic practices in milk and meat handling among a total of 186 respondents was determined by whether more than 50% of them (i.e., over 93 individuals) exhibited correct practices [33–35].

### 2.8. Data Collection Tools and Procedures

A structured standard questionnaire was developed after reviewing related literature. The questionnaire contained sociodemographic features and questions related to milk and meat handling practices. A different form of the questionnaire was developed for each group according to the character of their field/role. Questionnaire Translation Validation was done; that was the translation of English to the local language, Afan Oromo, done to ascertain that the translated instrument reflects the meaning and intent of the original. Back‐translating from Afan Oromo to English was also done by an independent forward translation translator. Comparing the transcription with the original and establishing differences to revise the translated instrument was done by a bilingual translator.

Pretesting of the questioner was done which aims to uncover potential flaws: clarity and understandability, relevance, and completeness, and operational details: ensuring flow, format, and ease of administration. Identification of any wordings or structural elements that may lead to biased answers was assessed. With our pretesting, we check the revision and enhancement of the instrument, enhancing its validity and reliability. During our pilot testing of the translated instrument on a small sample of the target population, we identified understanding and challenging or culturally sensitive items. The respondents who cannot read and write were interviewed face‐to‐face, whereas the educated respondents were allowed to read and fill out the questionnaire.

### 2.9. Data Analysis and Management

The raw data collected on the paper was checked for any mistakes and transformed into and organized in Microsoft Excel. The organized data transferred to the SPSS software Version 27 for analysis. The number and/or frequency of the respondent’s sociodemographic and milk and meat handling practices distributions were analyzed using descriptive statistics. Binary logistic regression was employed to determine the association between sociodemographic features and good hygienic milk and meat handling practice level as well as the *p* value and odds ratio at a 95% confidence interval or 5% significance level.

### 2.10. Ethical Consideration

The study was complied with the Declaration of Helsinki ethical principles for research involving human subjects. The ethical clearance committee at Bahir Dar University provided the required approval of this research proposal (Ref. No. 1/033/1‐1‐3) before conducting the research. Using a letter from Bahir Dar University, obtained permission from Ambo and Guder town management office demonstrated the approval of the work. During the interview, the purpose of the study was explained. After a thorough explanation of the objectives and relevance of the study, the procedure, benefits, and their rights, informed consent was obtained from the participants, and awareness was created. Data was collected after full informed verbal consent was obtained.

## 3. Results

### 3.1. Sociodemographic Characteristics of the Respondents

In this study, the total number of participants for all cases was 186 (80 milkers, 30 milk sellers, 44 abattoir workers, and 30 meat handlers) food handlers from both towns. The majority (112 (60.2%)) of the respondents were from Ambo town based on the size of its population. More than half of the respondents (106 (57%)), including all participants of the abattoir (46 (23.7%)) and meat sellers (30 (16.1%)), were males. The average age of the participants was 35.4 years, with a minimum of 18 and a maximum of 70 years. A slight majority of the participants (58 (31.2%)) had no formal education, while only 22 (11.9%) completed higher education. Almost all of the respondents (184 (98.9%)) had never taken any formal food handling training except for two (1.1%) meat inspectors. The overall sociodemographic of respondents was summarized in Table 1.

**Table 1:** Sociodemographic characteristics of the respondents (*n* = 186).

**Characteristics**		**Total no**	**Percentage**
Address	Ambo	112	60.2
	Guder	74	38.8

Gender	Male	106	57
	Female	80	43

Age	18–30	76	41
	31–43	70	37.5
	> 43	40	21.5

Educational level	Illiterate	58	31.2
	Primary	57	30.6
	Secondary	49	26.3
	Higher	22	11.9

Role of duty	Milkers	80	43
	Milk sellers	30	16.1
	Butchers	44	23.7
	Meat sellers	30	16.1
	Meat inspector	2	1.1

Food handling training	Yes	2	1.1
	No	184	98.9

Experience year	1–5 years	119	58.6
	6–10 years	42	22.6
	> 10 years	35	18.8

### 3.2. Milk Handling Practices of Milker’s

Of all 80 participants, 42 (52.8%) had a good level of milk handling practices. Only 24 (30%), 33 (41.2%), and 37 (46.2%) of the milkers used to milk their cows in a separate milking room, wash their cow’s udder before milking, and wash their hands before and in between milking, respectively. The majority of the milkers (61, 76.2%) did not wash the cows’ sleeping area or barns.

Of the milkers, 32 (40%) were wearing the personal protective equipment, with only three (3.8%) washing/changing daily. Nearly more than half of the milkers (43 (53.8%)) used locally available plants to clean and disinfect milk utensils. About 52 (65%) of the participants used water provided through a pipeline, whereas only 40 (50%) used it to wash their hands with soap (Table 2).

**Table 2:** Milk handling practices of milkers of the study area (*n* = 80).

**Variables**	**Category**	**Total no.**	**Percentage**
Management type	Small holder	61	76.2
	Farm	19	23.8

Having separate milking room	Yes	24	30
	No	56	70

Barn cleaning	Sweeping only	61	76.2
	Sweeping and washing	19	23.8

Frequency of barn cleaning	Daily	65	81.3
	Twice a week	4	5
	Three times a week	5	6.2
	Weekly	6	7.5

Sources of water	Pipeline	52	65.0
	River	18	22.5
	Both	10	12.5

Wear personal protective equipment	Yes	32	40
	No	48	60

Frequency of wash personal protective equipment	Daily	3	3.8
	Twice a week	16	20
	Three times a week	0	0.00
	Weekly	13	16.3

Time of wash hand	Before milking	43	53.8
	Before and between milking	37	46.2

Wash hand after sneezing or coughing	Yes	31	38.8
	No	49	61.2

Wash hand with	Water only	40	50
	Water + soap	40	50

Wash cow’s udder before milking	Yes	33	41.2
	No	47	58.8

Clean and disinfect milk utensils	Yes	53	66.2
	No	27	33.8

Disinfectants for utensils	Water + local plants + smoking	43	53.8
	Water + soap	37	46.2

Machine milking	Yes	1	1.2
	No	79	98.8

Refrigerate the milk	Yes	28	35
	No	52	65

Sell milk	Yes	37	46.3
	No	43	53.8

Mix morning and evening milk before sell	Yes	44	55
	No	36	45

Adulteration of milk	Water	12	15
	Sugar or other spices	0	0.00
	None	68	85

Process milk	Yes	43	53.8
	No	37	46.2

### 3.3. Milk Handling Practices of Milk Sellers

Among 30 milk sellers, only 13 (43.3%) had good milk handling practices. About 27 (90%) participants used water provided through the pipeline. Slightly more than half (16 (53.3%)) and 23 (76.7%) of participants used the water together with soap to clean utensils and wash their hands, respectively. The majority (18 (60%)) of the milk sellers used to wear regular clothes while working, and only two (6.7% of respondents) washed/changed personal protective wear every next working day. Slightly less than half (14 (46.7%)) of the milk sellers were used to smoking milk utensils; yet, none of the respondents used any chemicals to disinfect their utensils or tested the quality of milk using any instruments such as a lactometer (Table 3).

**Table 3:** Milk handling practices of milk sellers (*n* = 30).

**Variables**	**Category**	**Frequency**	**Percentage**
Sources of water	Pipeline	27	90
	River	0	0.00
	Both	3	10

Wear personal protective equipment	Yes	12	40
	No	18	60

Frequency of wash personal protective equipment’s	Daily	2	6.7
	Twice a week	9	30
	Three times a week	2	6.7
	Weekly	17	56.6

Wash hand after sneezing or coughing	Yes	10	33.3
	No	20	66.7

Wash hand with	Water only	7	23.3
	Water + soap	23	76.7

Clean and disinfect utensils	Water + local plant + smoking	14	46.7
	Water + soap	16	53.3
	Water + soap + alcohol	0	0.00

Adulteration	Sugar	20	66.7
	Sugar and water	10	33.3

Refrigerate the milk	Yes	7	23.3
	No	23	76.7

Processing milk	Yes	13	43.3
	No	17	56.7

Test milk quality	Yes	13	43.3
	No	17	56.7

Method of test milk quality	Sensory testing	13	43.3
	Lactometer	0	0.0
	None	17	56.7

### 3.4. Meat Handling Practices of Butchers and Meat Inspectors

From 46 participants, more than half of the abattoir workers (25 (54.3%)) had a good meat handling practice level. About 30 (65.2%) of the participants were wearing aprons, and only four (8.7%) of the butchers washed/changed them every next working day. Around three‐fourths (38 (82.8%)) of the butchers washed their hands after using the toilet; however, only 11 (23.8%) washed their hands after sneezing or coughing. Of the butchers, 34 (73.9%) used different knives for meat and offal, and 30 (65.2%) cleaned and disinfected the instrument every time before use (Table 4).

**Table 4:** Meat handling practices of abattoir workers (*n* = 46).

**Variables**	**Category**	**Frequency**	**Percentage**
Rest and fasting animals in lierage	Yes	46	100
	No	00	0.00

Frequency of clean/wash the abattoir	Daily	25	54.3
	Twice a week	00	0.00
	Three times a week	21	45.7
	Weekly	0	0.00

Sources of water	Pipeline	24	52.2
	River	0	0.00
	Both	22	47.8

Wear personal protective equipment	Yes	30	34.8
	No	16	65.2

Frequency of wash personal protective equipment	Daily	4	8.7
	Twice a week	17	37
	Three times a week	8	17.3
	Weekly	17	37

Wash hand before slaughter	Yes	21	45.7
	No	25	54.3

Wash hand after sneezing or coughing	Yes	11	23.9
	No	35	76.1

Wash hand after using toilet	Yes	38	82.6
	No	8	17.4

Use soap to wash hand	Water only	11	23.9
	Water + soap	35	76.1

Use different knives for meat and offal	Yes	34	73.9
	No	12	26.1

Clean and disinfect meat utensils	Yes	30	65.2
	No	16	34.8

Clean and disinfect by	Water only	16	34.8
	Water+ soap	30	65.2
	Water + soap + alcohol	0	0.00

### 3.5. Meat Handling Practices of Meat Sellers

From 30 respondents, half (15 (50%)) of the meat sellers had good meat handling practices. About 28 (93.3%) participants used water provided through the pipeline to wash utensils and prepare food. More than half (17 (56.7%)) and 18 (60%) of participants used the water together with soap to clean utensils and wash their hands, respectively. Slightly more than half (16 (53.3%)) of the meat sellers used to wear protective clothes while working, and only 7 (23.3%) of respondents washed/changed personal protective wear every next working day. Only two (6.7%) of the meat sellers had not washed their hands after using the toilet, whereas only 12 (40%) had washed their hands after sneezing or coughing. About 20 (66.7%) of the meat sellers had properly cleaned utensils and a meat storage area, yet none of the respondents used any chemicals to disinfect their utensils or tested the quality of meat using any instrument such as a thermometer (Table 5).

**Table 5:** Meat handling practices of meat sellers (*n* = 30).

**Variables**	**Category**	**Frequency**	**Percentage**
Sources of water	Pipeline	28	93.3
	River	0	0.00
	Both	2	6.7

Wear personal protective equipment	Yes	16	53.3
	No	14	47.7

Frequency wear personal protective equipment	Daily	7	23.3
	Twice a week	4	13.4
	Three times a week	7	23.3
	Weekly	12	40

Wash hand after sneezing or coughing	Yes	12	40
	No	18	60

Wash hand after toilet	Yes	28	93.3
	No	2	6.7

Use soap to wash hand	Water only	12	40
	Water + soap	18	60

Use different knives for meat and offal	Yes	24	80
	No	6	20

Properly clean meat storage area	Yes	20	66.7
	No	10	33.3

Clean and disinfect utensils and storage area	Water only	13	43.3
	Water + soap	17	56.7
	Water + soap + alcohol	0	0.00

Test meat quality	Yes	14	53.3
	No	16	46.7

Type of test meat quality	Sensory testing	13	43.3
	Thermometer	0	0.00
	None	17	56.7

Refrigerate the meat	Yes	16	53.3
	No	14	46.7

### 3.6. Overall Hygienic Meat and Milk Handling Practices

**Figure 3:**
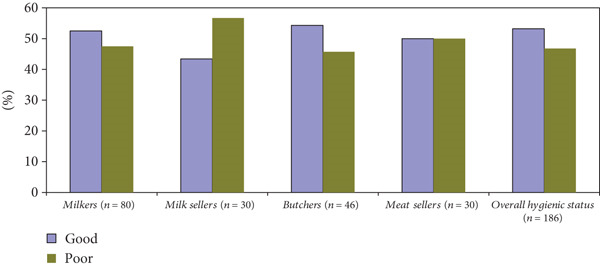
Overall hygienic milk and meat handling practices.

The current study revealed that 52.8%, 43.3%, 50%, and 54.3% of milkers, milk sellers, abattoir workers, and meat sellers had good practices in food handling practices, respectively. The overall hygienic status of the study area among the value chain is 53.2% (Figure 3).

### 3.7. Association Between Sociodemographic Characteristics and the Value Chain Practice Level

Even though milk and meat handlers from Ambo, aged between 31 and 43 (except milk sellers), and illiterate respondents showed good food handling practices, there was no statistically significant association between sociodemographic features and food handling practices (Table 6). Milkers and meat sellers’ years of experience had significant food handling practices (*p* value = 0.015). Milk sellers with ages between 31 and 43 were statistically significant practitioners (*p* value = 0.034). The status of butchers from Guder town showed statistically significant meat handling practices (*p* value = 0.001). The relationship between the sociodemographics of milk and meat handlers and their practice level is indicated in Table 6 and Table 7 below.

**Table 6:** The relationship between sociodemographic features and milk handlers.

**Sociodemographic features**		**Milker’s practices’ level**		**OR**	**(95% CI)**	**p** **value**	**Milk sellers’ practice level**		**OR**	**(95% CI)**	**p** **value**
		**Good**	**Poor**				**Good**	**Poor**			
Address	Ambo	27	23	1	Ref.		11	7	1	Ref.	
	Guder	13	17	2.54	(0.35–7.4)	0.135	9	3	0.2	(0.18–5.40)	0.65

Gender	Female	35	26	1	Ref.		8	3	1	Ref.	
	Male	5	14	0.31	(0.11–2.31)	0.089	12	7	1.7	(0.23–5.43)	0.31

Age	18–30 years	12	16	1	Ref.		12	3	1	Ref.	
	31–43 years	13	6	1.51	(1.28–16.5)	0.010	6	9	2.07	(1.21–5.70)	0.03
	> 43 years	15	18	3.61	(1.50–9.43)	0.006	2	4	1.5	(1.29–13.21)	0.04

Education level	Illiterate	20	19	1	Ref.	0.504	6	3	1	Ref.	
	Primary	8	18	0.2	(0.03–2.38)	0.218	6	4	2.0	(0.23‐5.43)	0.90
	Secondary	8	12	0.14	(0.03–5.68)	0.152	4	3	1.2	(0.11–4.76)	1.00
	Higher	4	1	0.15	(0.11–4.76)	0.145	4	0	1.0	(0.23–5.43)	1.00

Experience year	1–5 years	14	20	1	Ref.	0.033	8	8	1	Ref.	
	6–10 years	11	15	1.17	(1.01–9.82)	0.027	9	3	2.01	(1.11–4)	0.017
	> 10 years	15	5	1.13	(1.02–2.76)	0.013	3	0	2.33	(1.21–23.70)	0.01

Abbreviations: CI, confidence interval; OR, odds ratio; Ref., reference category.

**Table 7:** The relationship between sociodemographic features and meat handlers.

**Sociodemographic features**		**Butchers’ practices’ level**		**OR**	**(95% CI)**	**p** **value**	**Meat sellers’ practice level**		**OR**	**(95% CI)**	**p** **value**
		**Good**	**Poor**				**Good**	**Poor**			
Address	Ambo	19	5	1	Ref.		10	10	1	Ref.	
	Guder	6	16	2.7	(0.82–6.3)	0.001	5	5	0.88	(0.46–6.80)	0.90

Age	18–30 years	12	9	1	Ref.		4	6	1	Ref.	
	31–43 years	13	11	1.0	(1.31–7.95)	0.01	10	9	2.00	(0.82–6.3)	0.00
	> 43 years	0	0	1.32	(1.22–21.4)	0.00	1	0	1.21	(0.43–34)	0.22

Education level	Illiterate	0	0	1	Ref.		6	4	1	Ref.	
	Primary	8	12	2.1	(0.31–7.95)	0.07	7	8	3.91	(0.11–10.9)	0.53
	Secondary	14	4	1.25	(0.19–3.10)	0.27	1	2	1.23	(0.2–4.01)	0.92
	Higher	3	5	1.9	(0.81–21.60)	0.42	1	1	0.42	(0.08–4.7)	0.74

Experience year	1–5 years	13	4	1	Ref.		4	4	1	Ref.	
	6–10 years	14	5	1.19	(1.37–9.80)	0.03	4	10	1.33	(1.09–6.3)	0.06
	> 10 years	10	0	1.73	(1.10–5.44)	0.009	7	1	2.01	(1.11–10.9)	0.015

*N* *o* *t* *e*: N.B. with regard to gender, all meat handlers were males.

Abbreviations: CI, confidence interval; OR, odds ratio; Ref., reference category.

## 4. Discussion

Assessment of milk and meat handling practices involves evaluation of hygiene, storage, and processing practices in order to achieve food safety and quality. This involves looking into farm‐to‐consumer practices with a focus on contamination risks and compliance with regulations [4, 36, 37].

The study was the first of its kind to analyze the milk and meat handling practices in the belt areas of the Ambo and Guder towns, West Shewa Zone, Oromia, Ethiopia. In this study, 52.8%, 43.3%, 50%, and 54.3% of milkers, milk sellers, abattoir workers, and meat sellers had good practices on food handling, respectively. It indicates that the milk handling practices of milkers are 10% better than those of milk sellers. The overall hygienic status of the study area among the value chain is 53.2%. Our finding was less than the finding of Kassaw et al. [35]: 62.1% adhered to proper hygienic practices. The result of the current study showed that most of the respondents’ practice levels were arbitrarily at a good level. However, still nearly half of the respondents had poor practices of milk and meat handling. Thus, it is supposed that it needs real interventions that improve milk and meat handling practices [34].

### 4.1. Milking and Milk Handling Practices

Hygienic practices like washing udders before milking, cleanliness of milking equipment, and a clean milking environment are critical. Storage and cooling processes of milk, such as refrigeration, are necessary to prevent microbial growth and maintain quality [6, 36]. Processing, such as pasteurization or other processing methods, is used to further improve milk safety. Clean water is used for cleaning equipment and animals, as dirty water can be used as a source of pathogens. In addition, the equipment used for milking, storage, and transport, as well as how clean and appropriate for food handling they are, should be observed. Furthermore, the milking animals also need to be free from disease [4, 11, 37–39].

This study showed that 30% of the population conducts the milking in a separated milking room or area, and 41.2% used to wash the cow’s udder before milking. It is slightly two to four times better than the study revealed by Amenu et al. [40] in Borana, a pastoral community in Ethiopia, who reported 15.2% and 11.4%, respectively, and nearly two times worse than the study by Taddese et al. [39] in North Shewa Zone, Oromia, Ethiopia, and less than 58.5% reported according to Mekonen and Mengistu [41] around Bahir Dar, Ethiopia. The present study also revealed that 81.3% of the respondents clean the cow’s barn daily, which is much higher than the 51% and 49.2% reported by Mekonen and Mengistu [41] around Bahir Dar, Ethiopia, and Taddese et al. [42] in the North Shewa Zone, Oromia, Ethiopia, respectively.

The majority of the milkers (53.8%) have replied that they wash their hands only before milking, which is greater than the 21% reported by Amenu et al. [40] in Borana, Oromia, and less than the 75.8% reported by Taddese et al. [39] who washed their hands before milking and between milking two or more cows. This variation might be due to the production system followed by the population and the lifestyle differences in the study area. For example, in both Ambo and Guder towns, keeping dairy cows is urban and peri‐urban, and in this study, neither pastoralists nor pure smallholders participated.

The present study also reveals poor milking, handling, and storage practices (except for water) of milk, which agree with previous studies that suggest factors affect milk quality as reported by Yeserah et al. [14] in Bahir Dar city, Ethiopia, and Nyokabi et al. [6] in Ethiopia.

In this study, there is a gap between the milk equipment cleaning methods of the milker’s respondents and milk sellers by 53.8% and 46.7%, respectively, in using locally available plants and fumigation, which could be due to the interest from customers in the case of milk sellers. However, washing and smoking are normal practices (92.9%), according to a report by Yeserah et al. [14], Bahir Dar City, Ethiopia. Almost all milk sellers (90%) and 65% of milkers in this study used water provided through a pipeline and considered it clean water, which is less than reported by Yeserah et al. [14], Bahir Dar City, Ethiopia, and Taddese et al. [39] in North Shewa Zone, Oromia, Ethiopia, which are 24.5% and 26.8%, respectively.

### 4.2. Meat Handling Practices

Meat quality is induced mainly by contributing factors such as producers, traders, abattoirs, butchers, processors, and consumers [11, 43]. The present study indicated that 50% and 54.3% of the respondents working in butcher and meat seller (shop) places had good practices on food safety and hygiene, respectively. It is poorer and better, respectively, than the 42.4% and 68.6% reported by Gebremedhin et al. [42] at Ambo and Holeta Towns, Oromia Region, Ethiopia. It is much less than meat handlers (70%) Yenealem et al. [44], Gondar town, Amhara region, Ethiopia. This variation might be due to the respondent’s level of education, mainly of butchers, as in this study all had formal education. Moreover, 38.4% of abattoir workers and 53.3% of meat retailers practice wearing aprons and other personal protective equipment, which is lower than 48.3% and 61.9% in Ambo and Holeta towns by Gebremedhin et al. [42]. This is slightly agreed with the finding (42% and 48%, respectively) in Arbaminch town [43]. However, somehow it is supported by practices of meat handlers (31.7% and 36.3%) from Zemachu [45], Gedeo Zone, Southern Ethiopia.

In the current study, washing hands after visiting the toilet, both for abattoir men (82.6%) and meat shop retailers (93.3%), is almost normal, whereas they are less serious about washing hands after sneezing and coughing (23.9% and 40%), respectively. It was reported as very serious and worst in respondents from Yenealem et al. [44], Gondar town, Amhara region, Ethiopia. This gap may arise from the absence of food handling training for the respondents.

According to Gebremedhin et al. [42], meat contamination could occur due to different possibilities such as storing food in unclean utensils, holding food at a temperature that would allow microbial growth, utilization of poor‐quality water, using packaging materials that are not of food‐grade quality, a storage site that had no facilities for waste disposal, and utilization of unclean utensils. Besides, the lack of awareness in basic personal cleanliness and safe food handling of butchers increases the contamination of meat by microorganisms. In addition to these, the environment of the slaughterhouse, the floor of the retail outlet, the air in the outlet, working equipment, and the vehicle used for the transportation of the meat act as the external sources of contamination [11, 46]. Furthermore, the informal methods of meat handling and marketing meat by butcheries undermine meat quality and safety, which is shared by the other socioeconomic conditions of Ethiopia [45]. Generally, the result of the current study revealed that meat handler’s age and years of experience were found to be statistically significant (*p* < 0.05) and showed good meat handling practices. Similar to our findings, research carried out by Zemachu et al. [45] in Ethiopia’s Gedeo zone found that meat handler’s age, experience, and education level were strongly associated with a high degree of meat handling ability.

The above different studies in Ethiopia and our study should show that poor milk and meat hygiene practices are persistent; this might be due to a combination of infrastructural, economic, cultural, and regulatory factors [47]. During our study observation, the majority of slaughterhouses and milk collection sites lack minimum sanitation facilities, cold storage, and clean water supply, while rural households have no choice but to depend on open‐air markets without refrigeration, where hygienic handling is impossible. Economic constraints contribute to the difficulty, as farmers and traders will resort to quick sales instead of investing in clean equipment such as stainless steel milk cans or modern slaughtering machinery. Maintaining a cold chain is also too expensive, particularly in our study area with irregular electricity. Additionally, there are gaps in knowledge that remain wide, where little is known about zoonotic diseases and the relationship between hygiene and human health. Routine beliefs such as the understanding that milk boiled is less nutritious or of poorer quality are some of the factors why raw commodities are still applied. Food and culinary culture are also to blame since raw meat foods such as kitfo and tire siga, and raw milk is well entrenched in our study area food culture. Distribution of meat during festivities typically bypasses official mechanisms of inspection altogether. Weak compliance with existing laws also contributes to the issue; there are food safety laws, but enforcement is inconsistent, particularly in our study areas. Coordination among health, agricultural, and trade officials is often poor, so unofficial markets go largely unchecked [4, 6, 47–49].

The government of Ethiopia has introduced a range of national and regional food safety policies to tackle these issues. Food safety, quality, and labeling are governed under the Food, Medicine and Health Care Administration and Control Proclamation (No. 661/2009), which compels licensure and inspection of food processing and handling premises [50]. The Ethiopian Standards Agency (ESA) sets standards for milk, meat, and other animal products for hygiene, patterned from international standards such as the Codex Alimentarius, although its implementation varies. Under meat hygiene, national regulations for abattoirs require ante‐mortem and post‐mortem examination, but our study area’s slaughtering takes place in unlicensed abattoirs [51]. Oromia Regional government has additional regulations on slaughter practice, but enforcement is undermined by the practice of backyard slaughtering and open‐market sales in our study areas. In the realm of milk hygiene, draft dairy development policies promote pasteurization, cold chain investments, and cooperative milk collection centers, while the Dairy Development Strategy promotes farmer training and the development of infrastructure; yet, these types of initiatives are still dominated by men in our study area. Despite these guidelines, there are large gaps between practice and policy. Control is unenforceable due to the fact that there are hardly any under‐resourced inspectors, and the market structure—over 80% of milk and meat traded on informal, unregulated markets—binds control. Also, consumer demand for clean products is weak, offering little economic incentive to producers and vendors to comply with standards [49, 51, 52].

### 4.3. Limitation of the Study

The limitations of this work were that variables such as knowledge and attitude of the value chain participants have not been considered. In addition, microbiological testing to assess the level of contamination was not included in this study.

## 5. Conclusions

The current study revealed that only nearly half of the milkers, milk sellers, abattoir workers, and meat sellers had good practices in food handling. This indicated that overall milk and meat handling practices among the value chain in the study area were found to be unsatisfactory/poor. This is because portions of the participants were still not practicing personal and utensil hygiene, which leads to poor practices in milk and meat handling, causing a public health hazard. The multivariable analysis indicated that age and years of experience were factors for good milk and meat handling practices among respondents in the study area. Other variables like gender, role in the value chain they work in, education level, and food handling training were not associated with the value chain meat and milk handling practices.

Thus, formal education needs to be the regulatory standard for participation in milk and meat marketing value chains. Implementing of focused training programs for market vendors, milk collectors, abattoir workers, and smallholder farmers on safe dressing of carcasses, proper animal handling, hygienic milking, and detection of zoonotic diseases in animals is essential. There is a need for the development and enforcement of interventions that aim to improve food hygiene by ensuring standards that are harmonized with country food safety guidelines, increased inspection capability, periodic certificates of hygiene, and implementations. Milk and meat hygiene community education is also being promoted through radio broadcasts, drama/theater clubs, hygiene clubs in schools, social media campaigns, and coordination with religious leaders and senior members in society. Further microbiological studies on the public health and economic implications of poor handling practices with regard to foodborne infections in the study region are suggested.

## Conflicts of Interest

The authors declare no conflicts of interest.

## Author Contributions

Birhan Agmas Mitiku: conceptualization, methodology, resources, supervision, visualization, writing—original draft, and writing—review and editing. Beka Ararsa Desisa: conceptualization, formal analysis, methodology, and writing—original draft. All authors reviewed and approved the final manuscript.

## Funding

This study was supported by the Bahir Dar University, Ethiopia; ref. No. 554/08/024.

## Data Availability

All information is available upon reasonable request from the corresponding author.
